# Response to Acute Psychophysical Stress and 24-Hour Glycemic Control in Healthy Older People

**DOI:** 10.1155/2012/803864

**Published:** 2012-07-08

**Authors:** Loretta DiPietro, Catherine W. Yeckel, Andrei Gribok

**Affiliations:** ^1^The John B. Pierce Laboratory, Yale School of Medicine, New Haven, CT 06519, USA; ^2^Department of Epidemiology and Public Health, Yale School of Medicine, New Haven, CT 06519, USA; ^3^The Department of Exercise Science, School of Public Health and Health Services, The George Washington University, 817 23rd Street, NW, Washington, DC 20052, USA; ^4^Beltsville Human Nutrition Research Center, The United States Department of Agriculture, Beltsville, MD 20705, USA

## Abstract

We examined the relation between stress reactivity and 24 h glycemic control in 17 inactive, healthy older people (≥60 years) under both a novel psychophysical stress and a seated control condition. Plasma cortisol was measured over the course of the stress and recovery periods. Glycemic control was determined over the subsequent 3 h from an oral glucose tolerance test (OGTT) and over 24 h *via* continuous glucose monitoring (CGM). We observed significant (*P* < 0.05) elevations in perceived stress, cardiovascular activity, and peak cortisol response at 30 min (10.6 ± 3.1 versus 8.6 ± 2.6 **μ**g*·*dL^−1^, resp.) during the stress compared with the control condition; however, 3 h OGTT glucose and insulin responses were similar between conditions. The CGM data suggested a 30–40 min postchallenge delay in peak glucose response and attenuated glucose clearance over the 6 h following the stress condition, but these alterations were not statistically significant. Healthy older people may demonstrate minimal disruption in metabolic resiliency following everyday psychological stress.

## 1. Introduction

“Stress” is a common and adaptive component of our interaction with the environment [1], and *allostasis *refers to the body's ability to reestablish stability (i.e., homeostasis) when confronted by various environmental challenges through the activation of neural, neuroendocrine, and neuroendocrine-immune responses [2]. There is some evidence from animal models that aging *per se* alters allostatic ability, although the data in humans are inconclusive [3]. Nonetheless, older age is characterized by diminished physical capabilities (e.g., vision, strength, and reaction time), which may make normal everyday experiences and challenges (like driving an automobile or crossing the street) more stressful [4]. Thus, older people may have more frequent exposures to stressful situations, along with a compromised ability to respond appropriately to them. There are significant individual differences in how individuals cope with environmental challenges, however, and this may be due to the interaction of heredity, development, education, and life experiences [5, 6]. Furthermore, there is substantial heterogeneity with regard to patterns of aging [7] such that some older people appear more resilient than others to the physiological consequences of various environmental challenges. Resiliency to psychophysical stress therefore may be considered an important indicator of successful aging.

To date, much of the experimental study of stress reactivity and health has focused on cardiovascular function. Despite the epidemiologic data describing the association between adverse psychosocial factors and poor diabetes control [6], the harmful effect of acute psychological stress on metabolic control is often difficult to demonstrate experimentally [8, 9]. Moreover, the studies that have investigated the role of stress response in glycemic control have included primarily younger people [8, 10], or those with already established type 1 or type 2 diabetes [9], have used specific tasks or responses to natural disasters [11] that are not usually encountered on a daily basis by older people, and have only considered glycemic responses over 2-3 hours. The purpose of this study was to examine the relation between stress reactivity and glycemic control over 24 h in healthy older people, using a common “everyday life” stress challenge. We hypothesized that (1) older subjects would demonstrate elevated cardiovascular and hormonal responses to this challenge compared with a control condition and (2) an exaggerated stress response would result in significantly disrupted glycemic control in the short term (i.e., 3 h) but not over 24 hours. Due to the robust health status of this study sample, we proposed that alterations in metabolic control in the short term would be normalized over the remainder of the day. To our knowledge, these hypotheses have not been tested before in healthy people of any age using continuous glucose monitoring.

## 2. Methods

### 2.1. Study Subjects

Older (≥60 years) volunteers (*N* = 6 men and 11 women) were recruited by advertisement from community senior centers throughout the greater New Haven County. Eligible subjects were nonsmoking, and not taking beta-blockers, glucose-lowering medication, antidepressants, or reporting an alcohol intake >2 drinks/day. To eliminate the confounding influence of cardiovascular fitness on stress reactivity and on glycemic control, all subjects were reported to be inactive (<2 days/week of moderate-intensity physical activity lasting more than 10 min duration). Interested subjects first were administered a cognitive function screening using the nine-item Short Portable Mental Status Questionnaire [12]. All persons achieving a score of 5 or fewer correct answers on the SPMSQ were excluded from further study. Interested subjects meeting the cognitive function criterion then were given a screening oral glucose tolerance test (OGTT (75 g load)) to rule out undiagnosed type 2 diabetes. Those with a fasting plasma glucose concentration ≥110 mg*·*dL^−1^, a 2 h postchallenge measurement ≥140 mg*·*dL^−1^ or at least one other postchallenge ≥180 mg*·*dL^−1^ were excluded from further study. Eligible volunteers had all details of the study explained to them and signed a form indicating their informed consent. All protocols were approved by the Human Investigations Committee of the Yale School of Medicine.

### 2.2. Study Protocol

Each study subject underwent two separate study conditions: (1) the actual psychophysical stress challenge and (2) a seated control condition. The two conditions were randomly ordered and spaced approximately 2–4 weeks apart. Because depression may influence the relation between stress response and insulin sensitivity, subjects were administered the Center for Epidemiologic Studies-Depression (CES-D) [13] scale (a 20-item measure of the frequency of depressive symptoms over the past week) one week prior to their first study. The Perceived Stress Scale (PSS) [14] (a 10-item measure of perceived psychological stress over the previous month) was also administered at this time.

On the day of testing, subjects arrived at the Hospital Research Unit (HRU) of the Yale Center for Clinical Investigation (YCCI) at 8:00 AM in the fasted state. We chose to apply the stress stimulus in the fasted (rather than postprandial) state to avoid an anticipated wide range of postmeal glucose excursions in these older people, which could potentially mask any stress-related effects on glycemic control. Subjects were weighed and the abdominal circumference [15] was measured. Subjects then sat in a semirecumbent position for the placement of a catheter in an antecubital vein. A blood sample was drawn (15 cc) for the determination of basal glucose, insulin, and cortisol. At approximately 8:30 AM, the probe of the continuous glucose monitoring system (CGM) (MiniMed, Sylmar, CA, USA) was inserted subcutaneously in the abdominal wall and the CGM was calibrated while subjects sat quietly in the recumbent position for a 60 min equilibration period. Following the equilibration period, a baseline blood sample was drawn for the study substrates and stress hormones of interest. At about 9:30 AM, subjects then either sat quietly for 30 min reading or listening to music (seated control) or were given instructions about the tasks involved in the psychophysical challenge. Following the 30 min psychophysical challenge (or seated control condition), subjects sat quietly for another 30 min recovery period. Blood samples were taken every 15 min over the experimental (or control) and the recovery periods, and heart rate and blood pressure were measured continuously *via* an automated device (Colin Medical Instruments, Komaki, Japan), with recordings every 5 min. Interstitial glucose concentrations were also measured continuously during this time using CGM. Following the recovery period (~10:30 AM), subjects were allowed to void and were moved from the testing chair to an adjacent bed in order to perform the 3 h OGTT. Following the OGTT (~1:30 PM), subjects were fed a standardized lunch (60% carbohydrate; 20% protein; ~32 kcal*·*kg body weight^−1^/day), instructed on the home use of CGM and provided with a standardized evening meal before being released from the HRU. Subjects were instructed to rest quietly for the remainder of the afternoon and evening, to eat their evening meal at ~6:00 PM, and to retire by 10:00 PM. On the following morning, subjects were visited at home in order to obtain fasting blood samples of glucose and cortisol, as well as to remove the glucose sensor probe.

### 2.3. Psychophysical Stress Challenge

The automated psychophysical test (APT) (National Public Services Research Center, 1996) [16, 17] is a computerized series of *timed performance measures* such as simple reaction time, choice reaction, visual tracking, static and dynamic acuity, and information processing. The APT contains a battery of items that tests specific automobile driving-related perceptual and cognitive abilities developed by the National Public Services Research Institute. As we employed these driving-related tasks solely to elicit a stress response, we did not actually score their performance. Subjects were told, however, that we were testing their driving performance as part of a study on psychophysical abilities in older age and diabetes risk. To enhance the social evaluation component of the psychophysical challenge, performance was “monitored” with a shame video camera. Immediately after the challenge (or control), subjects rated their *level of perceived threat* using 8 visual analog (100 mm) scales.

### 2.4. Oral Glucose Tolerance Test

To measure transient disruptions in glycemic control following the stress challenge, a 75 g OGTT was performed according to the American Diabetes Association guidelines [18]. Blood samples (5 cc each) were collected before (−15, 0 min) and following (5, 10, 20, 30, 45, 60, 90, 120, 150, and 180 min) glucose ingestion for the determination of glucose and insulin concentrations. The OGTT was terminated when the glucose value for a given subject returned to be within 10 mg*·*dL^−1^ of baseline; otherwise subjects were monitored over 3 hours.

### 2.5. Continuous Glucose Monitoring

Whole-day interstitial glucose profiles were collected over 24 hours using the CGMS. The device provided glucose pattern and trend data up to 288 times per day over 24 h [19, 20] by measuring interstitial glucose and converting it at a glucose oxidase interface to hydrogen peroxide, which was then oxidized to produce an amperometric signal [19]. This signal is proportional to the interstitial glucose concentration and is stored in the monitor. The stored amperometric data then were transferred and converted to glucose concentrations after data collection was completed using an infrared link to a personal computer and the data analyzed using the CGM systems solution software (version 3.0B). Four to six actual blood glucose values (obtained *via* glucometer (Medtronics, Sylmar, CA, USA)) were entered into the monitor in order to calibrate the interstitial readings. In addition to continuous readings, a number of summary data over a 24 h period were generated by CGM: (1) averaged premeal; (2) averaged 2 h postprandial; (3) 24 h averaged glucose concentrations.

### 2.6. Ratings of Perceived Stress and Threat

Upon completion of the psychophysical challenge, subjects rated their level of perceived stress and threat to social self using 8 visual analog (100 mm) scales [3]. These scales assessed: (1) how difficult the challenge was; (2) how confident they were in their performance; (3) how much they were personally involved in the challenge; (4) how controllable the situation was; (5) how threatening the situation was; (6) how much stress they were experiencing due to failure; (7) how much stress they were feeling due to time constraints; (8) how content they were with their performance. At one end of the scale was the rating of “not at all” (0 mm) and at the other (100 mm), the rating “extremely.”

### 2.7. Stress Hormone and Substrate Analysis

All blood samples were analyzed in the core laboratory of the YCCI. Plasma *cortisol *concentrations were determined by radioimmunoassay (Diagnostic Products Corporation, Los Angeles, CA, USA). Plasma *glucose* was analyzed by the glucose oxidase method (YSI 2300, Yellow Springs Instruments, Yellow Springs, OH, USA). Plasma immunoreactive *insulin* concentrations were determined with a double antibody radioimmunoassay (Diagnostic, Webster, TX, USA).

### 2.8. Statistical Analysis

Univariate statistics (*χ* ± SD) were first generated on all study variables for descriptive purposes. Total area and incremental area under the cortisol, as well as under the OGTT glucose and insulin curves (AUC), were determined by the trapezoidal method. In addition, we considered peak cortisol response, as well as the basal cortisol concentrations taken in the morning before and the morning after the psychological challenge to use in combination with the cortisol response curve as an additional indicator of integrated stress response. Mean differences in the physiological response variables between the two conditions were tested using paired *t*-tests. The relation between stress response and disrupted glycemic control (AUC and CGM summary variables) was determined using analysis of variance (ANOVA) for repeated measures. The original CGM data then were transferred into StatLab and were smoothed using cubic splines with an empirically selected smoothing parameter. Individual and pooled CGM data curves were examined by study condition to determine the degree of variability of response between the two conditions. Statistical significance was set at an alpha level of 0.05.

## 3. Results

**Figure 1:**
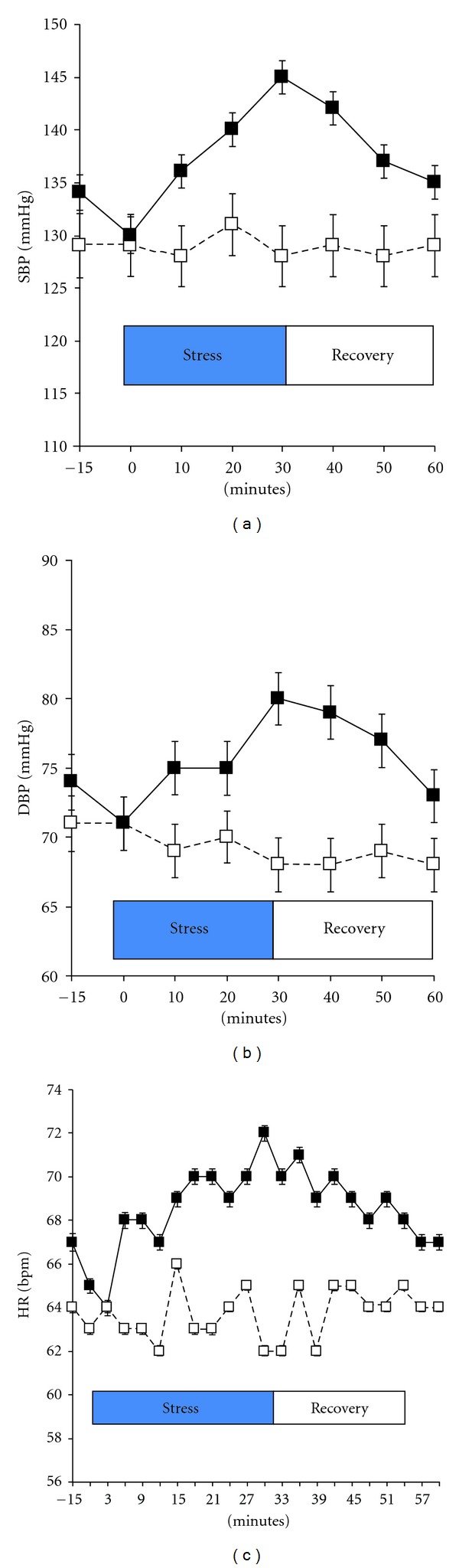
Differences in (a) systolic and (b) diastolic blood pressure and (c) heart rate response during the stress challenge (solid line) and control (broken line) condition. Data are mean ± se; *N* = 17.

The age of the study sample was 72 ± 9 years, with a range from 60 to 88 years. On average, subjects were overweight (27 ± 4 kg*·*m^−2^), but were normotensive (127 ± 10/73 ± 10 mmHg) and had normal glucose tolerance based on 2 h postchallenge blood glucose concentrations from the screening OGTT (130 ± 22 mg*·*dL^−1^). In addition, scores of depressive symptoms (9 ± 4) and perceived stress (12 ± 6) were within normative values for that age group (12,13). Not surprisingly, subject perception of the difficulty (56.8 ± 18.6 versus 3.7 ± 4.2 mm), threat (34.3 ± 30.3 versus 4.8 ± 5.7 mm), time stress (61.8 ± 39.4 versus 16.4 ± 28.0 mm), and failure stress (61.7 ± 34.3 versus 5.0 ± 5.8 mm) were significantly greater following the psychophysical challenge, compared with the control condition (*P* < 0.01). These perceptual data were corroborated by significant differences in cardiovascular responses in both systolic and diastolic blood pressure and heart rate between the two conditions (*P* < 0.01; Figure 1).

**Figure 2:**
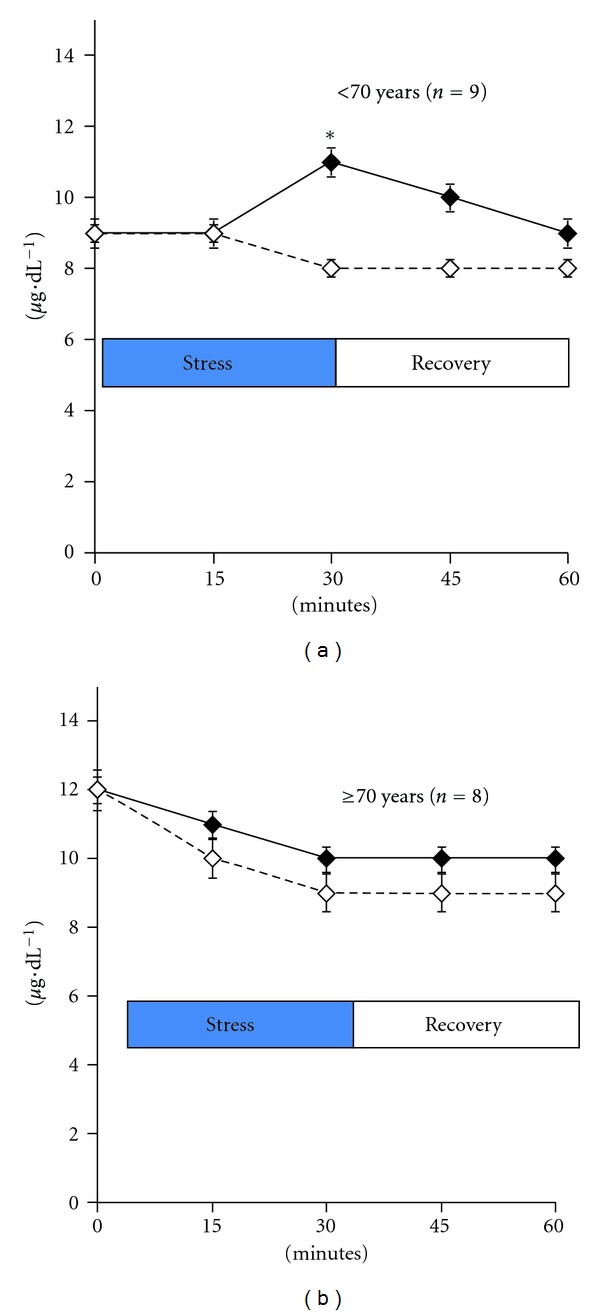
Cortisol responses between the stress condition (solid line) and the control (broken line) condition by age group. Age groups are based on the median cut point of the distribution. Data are mean ± se; *N* = 17; *P < 0.05.

Average cortisol AUC response over the experimental and recovery period was not significantly higher during the stress relative to the control condition, with the exception of peak response at 30 min (10.6 ± 3.1 versus 8.6 ± 2.6 *μ*g*·*dL^−1^, resp.; *P* < 0.05). When the data were stratified by median age, however, peak cortisol response from the stress condition was significantly amplified in subjects <70 years (*n* = 9) but reversed in those ≥70 years (*n* = 8) (Figure 2). For example, during the stress condition, 30 min cortisol concentrations increased from baseline by 22% (*P* < 0.05) in the younger subjects, but *decreased* by 7% in those aged 70 years and older. This variation in cortisol response was attributable to a significantly higher basal level in those ≥70 years, compared with their younger counterparts (*P* < 0.05).

**Figure 3:**
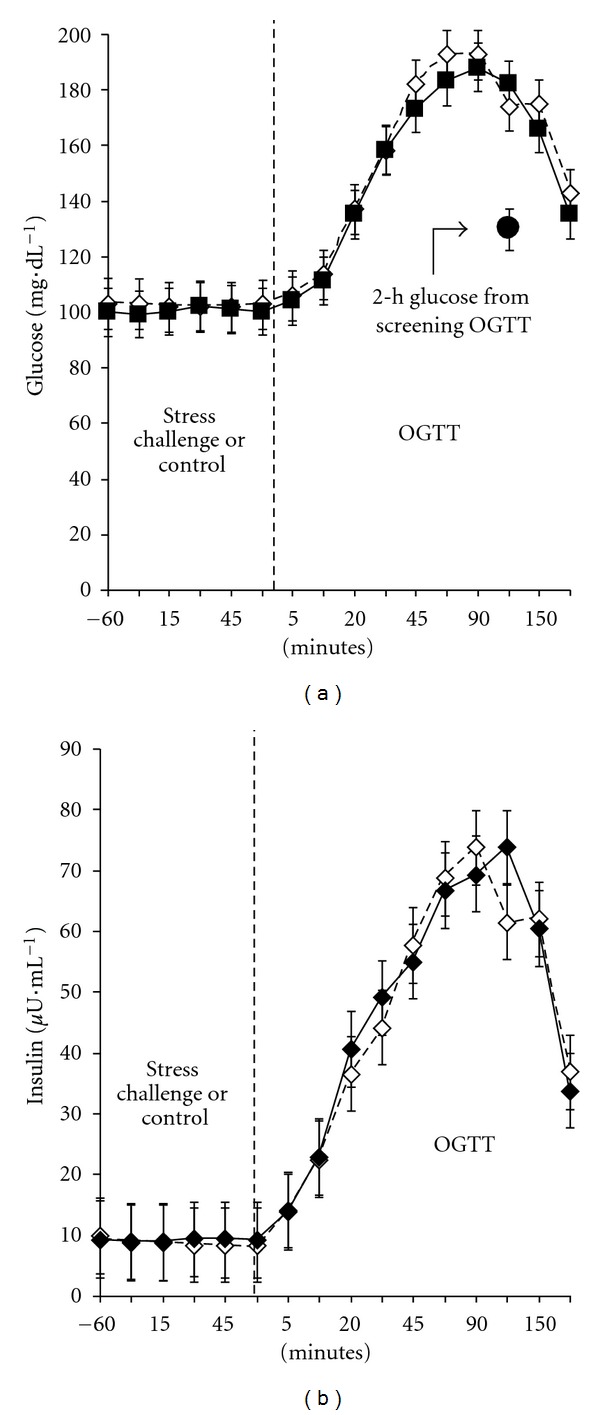
Glucose and insulin responses over the experimental period and the OGTT between the stress (solid line) and control (broken line) conditions. Data are mean ± se; *N* = 17. To convert to the International System of Units (mmol), multiply glucose values by 0.055.

**Figure 4:**
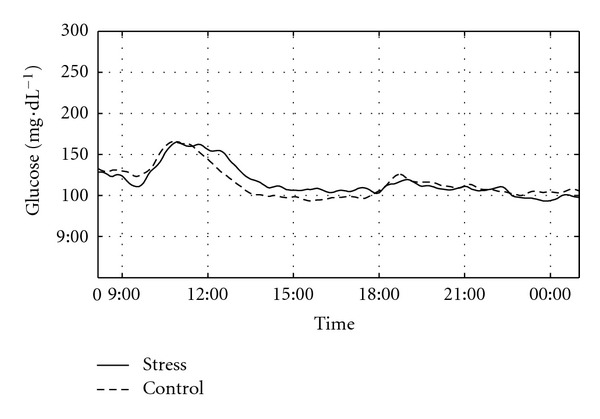
Glucose response curves obtained over 24 h *via* CGMS following the stress (solid line) or control (broken line) conditions. Data are mean values; Time = time of day; *N* = 17. To convert to the International System of Units (mmol), multiply glucose values by 0.055.

The OGTT-derived glucose and insulin response curves following the stress and control conditions are shown in Figure 3. Although clearly elevated and indicative of impaired glycemic control, the curves are quite similar for the two conditions, with the exception of the peak glucose response shifting from 60 to 90 min and the peak insulin response shifting from 90 to 120 min following the stress condition. These findings were unaltered after stratifying the data by age group (<70/≥70 yrs). Of note in this figure, however, is the average 2 h glucose concentration from the screening OGTT, which is significantly lower (130 ± 22 mg*·*dL^−1^; *P* < 0.05) than 2 h values following either the stress (182 ± 43 mg*·*dL^−1^) or control (174 ± 60 mg*·*dL^−1^) conditions. Glucose curves obtained *via* CGM over 24 h are displayed for each condition in Figure 4 and suggest a rightward shift and a 30–40 min delay in peak postchallenge glucose response following the stress condition, with glucose levels elevated relative to the control condition for as long as 6 hours afterward. We observed no difference, however, in averaged 24 h glucose concentrations (111.7 ± 12.3 versus 114.0 ± 24.0 mg*·*dL^−1^), or averaged prelunch concentrations (130.7 ± 43.9 versus 129.7 ± 47.0 mg*·*dL^−1^) between the two conditions, although averaged 2 h postlunch glucose concentrations (measured at approximately 4 PM) were slightly higher following the stress compared with the control condition (106.6 ± 22.3 versus 96.9 ± 12.3 mg*·*dL^−1^, resp.; *P* < 0.07). Variability of glycemic response appeared greater following the stress, compared with the control condition; however, differences in the estimates of mean skewness (0.70 ± 0.51 versus 0.57 ± 0.56, resp.) and kurtosis (3.38 ± 0.91 versus 3.04 ± 1.22, resp.) were not statistically significant.

## 4. Discussion

Adaptation to stress frequently involves the activation of the hypothalamic pituitary adrenal (HPA) axis in order to mobilize energy stores. Although the anti-insulin and gluconeogenetic actions of cortisol appear consistent with this notion, there is limited experimental evidence to support this idea [8]. We observed significant evidence of perceived stress and cardiovascular engagement to a 30 min psychophysical stressor among healthy older people, although HPA response appeared somewhat blunted relative to responses observed in other studies of older people [21, 22]. We did observe that our oldest study subjects (i.e., ≥70 years) had significantly greater basal cortisol concentrations compared with younger (<70 years) subjects, which is consistent with what has been proposed by Seeman and Robbins [23] and perhaps reflects the allostatic burden of aging, as the perceived stress scores were significantly higher in the oldest subjects compared with the younger subjects (14.4 ± 5.2 versus 9.5 ± 5.8; *P* < 0.05). Contrary to what we hypothesized, 3 h post-OGTT glucose and insulin concentrations were elevated equally under both conditions, suggesting little effect of the stress challenge *per se* on short-term glycemic control in those both older and younger than 70 years. Moreover, the CGM data provided evidence that tight glucose homeostasis may be only minimally altered following a stressful episode in healthy older people. Any subtle differences in glycemic control apparent up to 6 h after challenge had completely dissipated by 24 h, and CGMS data averaged over 24 h indicated no differences in overall response following the stress and control conditions. Whether these findings are indicative of heightened resiliency in successful (relative to normal) aging, of normal aging-specific homeostatic control, or of methodological differences between our study and others is not clear.

In general, studies that applied stressors during the fasted state observed significantly blunted cortisol and negligible glycemic responses [8, 9] compared with stress applied in the postprandial state [8, 9, 24], suggesting that ready access to energy is necessary for the permissive effects of HPA reactivity on glycemic control. We applied the psychophysical stress in the fasted state (thinking that prevailing glucose stores would be ample in this older population and wishing to avoid large postmeal glucose excursions), which more than likely explains the blunted cortisol response and minimal stress effects on 3 h postchallenge metabolic control.

Interestingly, 2 h postchallenge glycemic responses to the OGTT (measured at ~12:30 PM) were markedly elevated in both the stress and control conditions, compared with the early morning screening OGTT (182 ± 43 versus 174 ± 60 versus 130 ± 22 mg*·*dL^−1^, resp., for the stress, control, and screening conditions). These previously reported late-morning exaggerated glycemic excursions [25] may be attributed to the diurnal drop in insulin secretion reported in older people at this time of day [26] and more than likely prevented any further stress-related disruption. However, there is also other evidence that a stress challenge does not increase overall glycemic response, but rather shifts the peak response to the right, suggesting a stress-related *delay* in glucose absorption by the gut [23]. Our postchallenge glucose and CGM response curves are consistent with this delayed absorption phenomenon, as are the prevailing insulin concentrations from the OGTT, as the peak insulin response shifted from 90 min to 120 min. Nonetheless, if we consider the OGTT as the trigger for setting the stress-induced allostatic control response in motion, we observed only slight alterations with this control following the stress compared with the control condition—evidenced by the small lag in peak glucose response at 30–40 min after challenge, the slower rate of glucose clearance over the subsequent 6 hours (but not beyond), and the slightly greater individual variability in glycemic control. Consistent with HPA reactivity studies performed in the postprandial state [8, 9, 24], greater fuel availability 2 h following refeeding may have contributed to greater averaged postlunch glucose concentrations following the stress relative to the control condition, despite the fact that prelunch values were similar. These subtle differences would not have been detected without the use of CGM over 24 h.

The issue of selective survival is an important consideration when performing challenge studies in people who are in their 8th and 9th decades of life. Indeed, one hallmark of successful aging is a greater physiological resiliency to various environmental perturbations compared with people who have not survived to that age or who could not meet inclusion criteria for the study. Thus, although we used a stressor that mimics challenges often encountered daily in real life, it may not have been of sufficient intensity or duration for such a robust older population. This issue of an insufficient stimulus may be particularly problematic given the degree of inter- and intraindividual variability in physiological response to stress that was evident in our data and those of others [21, 27]. Also, the stress challenge was applied in the morning, when glucose concentrations were at their lowest. Had we performed the stress challenge in the later afternoon, when cortisol concentrations reach their daily nadir, we likely would have observed a greater relative increase in HPA response as others have [21, 22]. However, due to a possible diurnal drop in insulin secretion in the late afternoon, glucose responses under both conditions would have been exaggerated even more than we observed, thereby further masking any stress-induced effects.

In conclusion, the true nature of the relationship between stress and glucose homeostasis remains elusive and may be influenced by a number of individual, environmental, or temporal conditions. Our use of CGM allowed us to unmask some subtle short-term stress-related disruptions, but these alterations dissipated over 24 h in this healthy sample. On the other hand, given the number of environmental (e.g., driving, shopping) and psychological stressors encountered daily among the general population of older people, these findings may have greater relevance for less robust people with already-existing impairments in glucose control or with diabetes.
